# Music induced happy mood suppresses the neural responses to other’s pain: Evidences from an ERP study

**DOI:** 10.1038/s41598-017-13386-0

**Published:** 2017-10-12

**Authors:** Jiaping Cheng, Can Jiao, Yuejia Luo, Fang Cui

**Affiliations:** 1 Shenzhen Key Laboratory of Affective and Social Cognitive Science, Shenzhen University, Shenzhen, China; 2 College of Psychology and Sociology, Shenzhen University, Shenzhen, China; 3 Shenzhen Institute of Neuroscience, Shenzhen, China; 4 Faculty of humanities and social science, City University of Macau, Macau, China

## Abstract

In the current study, we explored the time course of processing other’s pain under induced happy or sad moods. Event-related potentials (ERPs) were recorded when participants observing pictures showing others in painful or non-painful situations. Mood induction procedures were applied to the participants before the picture observation task. Happy and sad moods were induced by listening to about 10 minutes of music excerpts selected from the Chinese Affective Music System (CAMS). The ERP results revealed that the induced mood can influence the early automatic components N1, P2, and N2 but not the later top-down controlled components P3 and LPP. The difference of amplitudes elicited by painful and non-painful stimuli was significantly different only in a sad mood but not in a happy mood, which indicates that comparing to a sad mood, the participants’ ability to discriminate the painful stimuli from the non-painful stimuli was weakened in a happy mood. However, this reduction of sensitivity to other’s pain in a happy mood does not necessarily reduce the tendency of prosocial behaviors. These findings offer psychophysiological evidences that people’s moods can influence their empathic response towards other’s pain.

## Introduction

Empathy is defined as the ability to vicariously share the affective states of others^1,2^. Neuroimaging evidences suggest that there are two components of empathy subserved by distinct brain networks^3^: the affective component reflecting rapid bottom–up activation of subcortical/cortical circuitries^4–7^ and the cognitive component that can be influenced by higher-level, top–down, signals originating from the prefrontal cortical circuitries^3,5^. Correspondingly, from the temporal aspect, empathy for pain involves two distinct processes as well. The first is an early automatic process resulting in emotional contagion and affective sharing. Secondly, there is a later controlled cognitive process that regulates empathic responses and makes a clear self-other distinction^8–12^. That is, the N1, P2 and N2 components are thought to reflect early, bottom-up processes involved in affective sharing, whereas the P3 and LPP components are thought to reflect a later, top-down process of cognitive evaluation^13,14^. A recent ERP study reported that painful expressions selectively modulated the early activity at 110–360 ms over fronto-central and centro-parietal regions, whereas painful contexts selectively modulated the late activity at 400–840 ms over these same regions, which also favor a model assuming distinct neural paths of perceptual and cognitive processing for other’s pain^15^.

Empathy is one of the hallmarks of psychological maturity that allows one to understand other people’s feelings and emotions, which is very important for social interactions^16^. Although “putting oneself into other’s shoes” seems to be a sophisticated ability for most human beings, our empathic responses are not always as accurate and sensitive as we believe they are. For example, if we feel secure, we tend to discount the idea that other people are anxious^17^. Our empathic response to other’s emotions and feelings can be strongly biased by our own emotional states and experiences. In one study, participants were asked to read a description of three hikers lost in the mountains without food or water, to predict whether hunger or thirst would be more distressing to the hikers. This study involved two groups of participants: those who hadn’t started exercising yet and those who had just finished a 20-minutes work-out. Results showed that compared to participants who hadn’t begun exercising, the participants who had just finished exercising were more likely to rate thirst as more unpleasant than hunger because they were hotter and thirstier themselves^18^.

Mood refers to an emotional state, which is less specific, less intense than feelings and emotions. Moods are typically described as having either a positive or negative valence^19,20^. The existing literature suggests that induced moods are able to influence the way we process the complex environment^21^. Numerous studies showed that the induced moods can modulate both of the cognitive and emotional processes, such as the participants’ level of self-focused attention^22–25^, visual perception strategies^26,27^, the processing of emotional words and pictures^28^. Moreover, the induced mood has also been found to be able to modulate social emotional behaviors such as moral judgment^29^ and decision making during the Ultimatum Game^30^.

We, therefore, assume that among the factors that may bias our empathic response, the observer’s current mood might be influential. However, few studies have explored what effect the moods of the observers may have on their empathic responses to others’ pain. Except one study found that participants who underwent a happy mood induction showed marginally higher empathy scores over those underwent the sad mood induction^31^. The lack of statistical significance and limited research technique (only questionnaires were used) suggest that more work is needed to determine what effect the moods have. The current research was designed to investigate how the induced mood influences the empathic response to other’s pain. Here, music was used to induce moods because the previous studies found that as compared with mood induced by films or images, the music-induced mood has the greatest personal relevance and was thought to have the greatest impact on other processes^30,32^. After the music mood induction, participants were instructed to passively observe pictures depicting a person’s hands/forearms/feet in painful or non-painful situations. ERPs during the observation of pictures under different induced mood were compared. Half of the participants listened to musical excerpts prepared to induce the happy mood first and the other half of the participants listened to musical excerpts prepared to induce the sad mood first. All participants went through both of the happy session and sad session. Four conditions were generated accordingly: observing painful pictures in a happy mood (*P_H*); observing non-painful picture in a happy mood (*NP_H*); observing painful pictures in a sad mood (*P_S*) and observing non-painful picture in a sad mood (*NP_S*).

Previous studies found being in a positive/negative mood makes emotion incongruent information less accessible and less intensive^33^. For instance, in one study, participants who were induced to trigger different moods were presented with computerized 100-frame movies in which the first frame always showed a face expressing a specific emotion (e.g. happiness). The facial expression gradually became neutral over the course of the movie. Participants were asked to indicate the frame at which the initial expression was no longer present on the face. Results show that emotion incongruent expressions were perceived to persist shorter than the emotion congruent expressions^34^. This “mood congruent effects” were also found in clinical populations such as depression, as this population demonstrated an increased likelihood of perceiving negative emotions in others and a decreased likelihood of perceiving positive emotions in others^35,36^. Other’s pain is a very salient negative stimulus which can trigger empathic pain and unpleasant feelings in the observers^4–6^. Based on the “mood congruent effects” hypothesis, we predicted that participants would be more sensitive to other’s pain in a negative mood. The painful stimuli and non-painful stimuli can be better distinguished under a sad mood than under a happy mood. In the psychophysiological neural markers of empathy for pain, there were five components that were consistently revealed in the comparison between the painful and the non-painful stimuli: N1, P2, and N2 components reflecting the early, automatic affective sharing process and P3 and LPP component reflecting the later, top-down controlled cognitive evaluation process^8–12,37^. We hypothesized that if the mood of the observer modulated the early, automatic stage of empathy, it should be evident in the N1, P2 and/or N2 component. Conversely, if the voluntary, top-down processing is influenced by different moods, then it should be evident in P3 and/or LPP.

## Methods and Materials

### Participants

Twenty-nine right-handed participants with no history of neurological disorders, brain injuries or developmental disabilities participated in the experiment. All of them have normal or corrected to normal vision. All participants signed an informed consent form before the experiment. The experiment was conducted in accordance with the Declaration of Helsinki and was approved by the Medical Ethical Committee of the Medical School of Shenzhen University, China. Three participants’ data were rejected due to intensive head movements during EEG recording (over 20% bad epochs). Finally, 26 participants’ data were included (14 male, 19.85 ± 0.66 years (mean ± S.E)).

### Stimuli

#### Auditory stimuli

In the current study, to induce happy and sad moods, we selected 20 pieces of music from the Chinese Affective Music System (CAMS), which was a standard set of music stimuli for emotional research with Chinese participants^38^. In the 20 pieces of music used in the current study, half of them were supposed to induce a sad mood while the other half were supposed to induce happy mood. The 10 happy music excerpts were significantly higher than the sad excerpts in the rating of happiness and arousal (Happiness: 6.93 ± 0.30 and 3.55 ± 0.73, *p* < 0.001; Arousal: 6.89 ± 0.40 and 4.05 ± 0.66, *p* < 0.001). No significant difference was found in the rating of expression and length (Expression: 5.35 ± 0.54 and 5.02 ± 0.64, *p* = 0.652; Length: 62.50 ± 4.43 s and 61.9 ± 4.79 s, *p* = 0.103).

#### Visual stimuli

**Figure 1:**
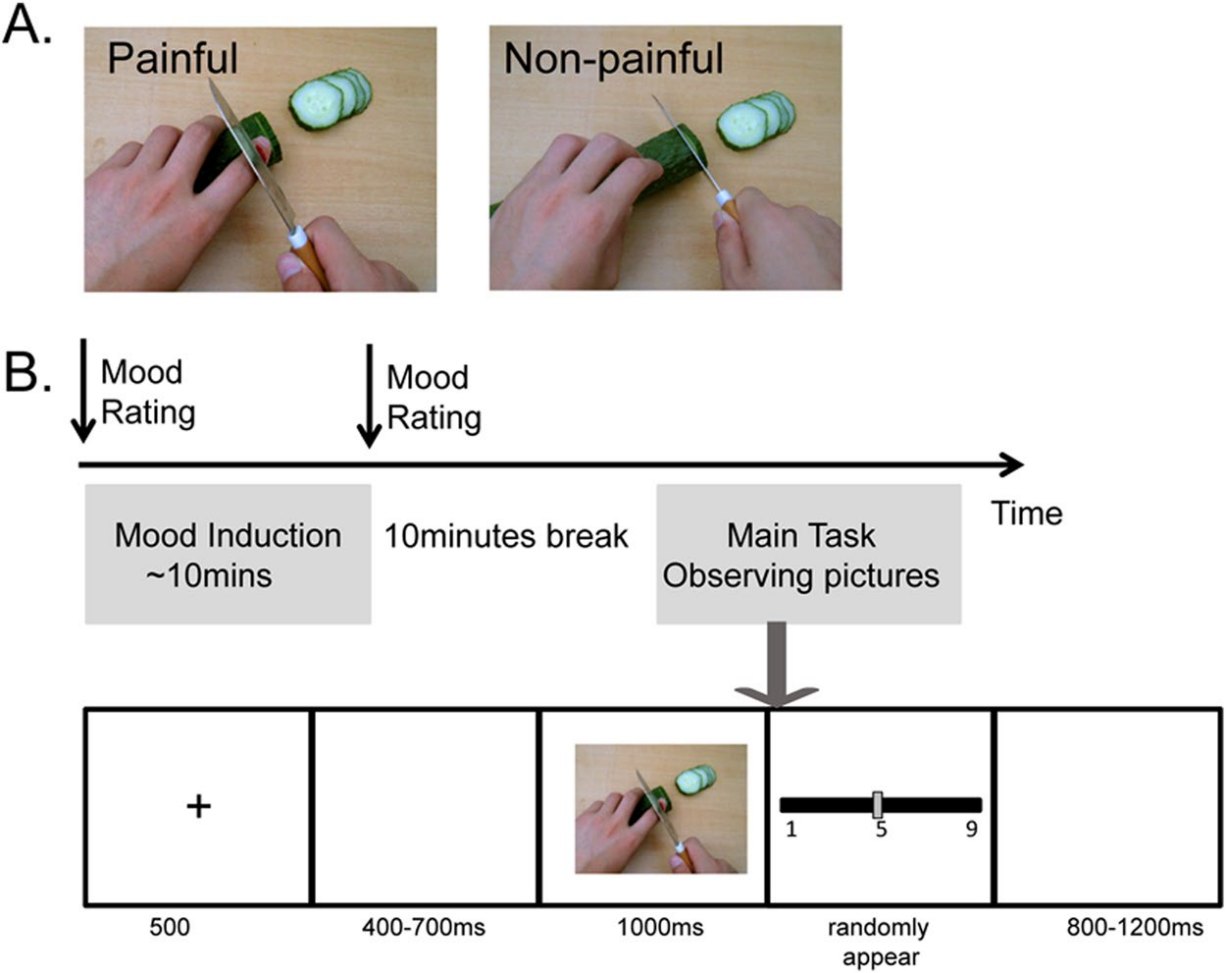
Examples of picture stimuli (**A**) and Procedures of the experiment (**B**).

The visual stimuli used in the experiment were pictures showing a person’s hands/forearms/feet in painful or non-painful situations, which have been used in previous ERP studies^39^. All the situations depicted in these pictures were ordinary events in daily life. All the events showing in the non-painful pictures were corresponding to those in the painful pictures, but without the nociceptive component (Fig. 1A). There were 60 painful pictures and 60 non-painful pictures in total. All of them had the same size of 9 × 6.76 cm (width × height) and 100 pixels per inch. Luminance, contrast, and color were matched between painful and non-painful pictures. Previous studies have confirmed that the painful and non-painful pictures were significantly different on the dimensions of pain intensity and emotional valence, according to the self-reported rating.

### Experimental procedures

Stimulus display and behavioral data acquisition were conducted using E-Prime software (Version 2.0, Psychology Software Tools, Inc, Boston, USA). During the task, participants sat comfortably in an electrically shielded room approximately 90 cm from a 15-inch color computer screen.

The experiment included two sessions: the “happy” session and the “sad” session. Half of the participants were assigned to run the happy session first and the other half were assigned to run the sad session first. To avoid the influence from the previous session, the participants were instructed to finish 10 mathematics questions (Two digit multiplication) between two sessions. In the start of each session, the participants were instructed to rate their initial mood on a 9-point scale (1: extremely unhappy to 9 = extremely happy). Then they would listen to the 10 happy/sad music excerpts continuously for about 10 minutes (each last ~1 min). After the music, they would be asked to rate their mood again on the same scale.

After the mood induction procedure, the participants were asked to observe pictures passively. In each trial, a fixation was presented for 500 ms, followed a 400 to 700 ms blank interval. Then the picture would present for 1000 ms. The ISI between trials was 800 to 1200 ms (Fig. 1B). Each session contains 120 trials, including 60 painful pictures and 60 non-painful pictures evenly. Each picture repeated twice in one session. The participants were informed that they would be asked questions about the pictures they observed during the task after the recording in order to keep their attention during the passive observation. Participants were asked three questions after the recording about the pictures they observed during the task: 1) did you see a person ignited the lighter and burned his hand? (Correct answer: Yes); 2) did you see a person put his hand into the boiling oil pan (Correct answer: Yes); 3) did you see a person cut his hand when he cut the meat (Correct answer: No).

Besides, in each session, there were 10 painful pictures and 10 non-painful pictures would be followed by a question randomly. The question was “how much do you want to help the person in the picture?”. The participants were instructed to rate their willingness of help on a 9-point scale (1: I don’t want to help him/her at all to 9: I do want to help him/her very much). The participant would press 1 to 9 on a keyboard to give their response.

### EEG acquisition and analysis

Electroencephalography (EEG) data were recorded from a 63-electrodes scalp cap using the 10–20 system (Brain Products, Munich, Germany). The channel TP10 was used as the reference during recording. Two electrodes were used to measure the electrooculogram (EOG). EEG and EOG activity was amplified at 0.01 Hz ~ 100 Hz band-passes and sampled at 500 Hz. All electrode impedances were maintained below 5 kΩ. EEG data were pre-processed and analyzed using MATLAB R2011b (MathWorks, US) and EEGLAB toolbox^40^. EEG data at each electrode were down-sampled to 250 Hz. The data were re-referenced to the common average. Then the signal passed through a 0.01–30 Hz band-pass filter. Time windows of 200 ms before and 1000 ms after the onset of the picture were segmented from EEG and the whole epoch was baseline-corrected by the 200 ms time interval prior to target onset. EOG artifacts were corrected using an independent component analysis (ICA)^41^. Epochs with amplitude values exceeding ± 50 µV at any electrode were excluded from the average.

Further statistical analysis was conducted in IBM SPSS Statistics 22 (IBM Corp., Armonk, NY, USA. Previous studies using similar stimuli suggested that the early components N1, P2, N2 and later component P3 and LPP were particularly related to observing other’s pain^11^. Analyses were conducted over the peak of the amplitude of N1, P2 and N2 component (peak to baseline) and the mean amplitudes of P3 and LPP component. According to the topographical distribution of the grand-averaged ERP and the literature^42,43^, five sets of electrodes from the frontal (FC3, FCz, FC4), central (C3, Cz, C4), centra-parietal (CP3, CPz, CP4), parietal (P3, Pz, P4) and parieto–occipital (PO3, POz, PO4) regions were chosen. Mean amplitudes were obtained from waveform averaged for all selected electrodes within each region. Repeated measures ANOVA (2 (Mood: Happy/Sad) × 2 (Picture: Painful/Non-Painful) × 5 Regions (frontal, central, centra-parietal, parietal, parieto-occipital) were performed for each component within its most pronounced time windows (N1 (100–160 ms), P2 (160–220 ms), N2 (200–300 ms), P3 (300–400 ms) and LPP (450–650 ms)). Degrees of freedom for F-ratios were corrected according to the Greenhouse–Geisser method. Statistical differences were considered significant at *p* < 0.05; post-hoc comparisons were Bonferroni-corrected at *p* < 0.05.

## Results

### Self-reported measures

Participants were asked to rate their mood before and after the mood induction procedure. Two paired t-tests were conducted between the ratings of self-mood before and after the mood induction for happy and sad mood separately. Before the mood induction, the difference between the happiness of self-reported mood was insignificant (Happy_Before: 5.39 ± 0.21; Sad_Before: 6.00 ± 0.18, t (25) = −1.96, *p* = 0.06). After the mood induction, the difference between the happiness of self-reported mood became significant (Happy_After: 6.77 ± 0.24; Sad_After: 4.91 ± 0.31, t (25) = 3.84, *p* = 0.001). The accuracy of the responses to the question after the recording was 91.03 ± 1.78% (mean ± sd), indicating they did observe the pictures carefully during the task.

For the rating of the willingness of helping, the ratings were compared using a 2 (Mood: Happy/Sad) × 2 (Picture: Painful/Non-Painful) repeated measured ANOVA. We found a significant main effect of Picture (F (1, 25) = 36.299, *p* = 0.01, η_p_^2^ = 0.59). Participants were more willing to help others in the painful situation than in the non-painful situation (6.71 ± 0.20 and 4.69 ± 0.31). The main effect of Mood and interaction of Mood × Picture were insignificant (*ps* > 0.062).

### ERPs

#### N1

**Figure 2:**
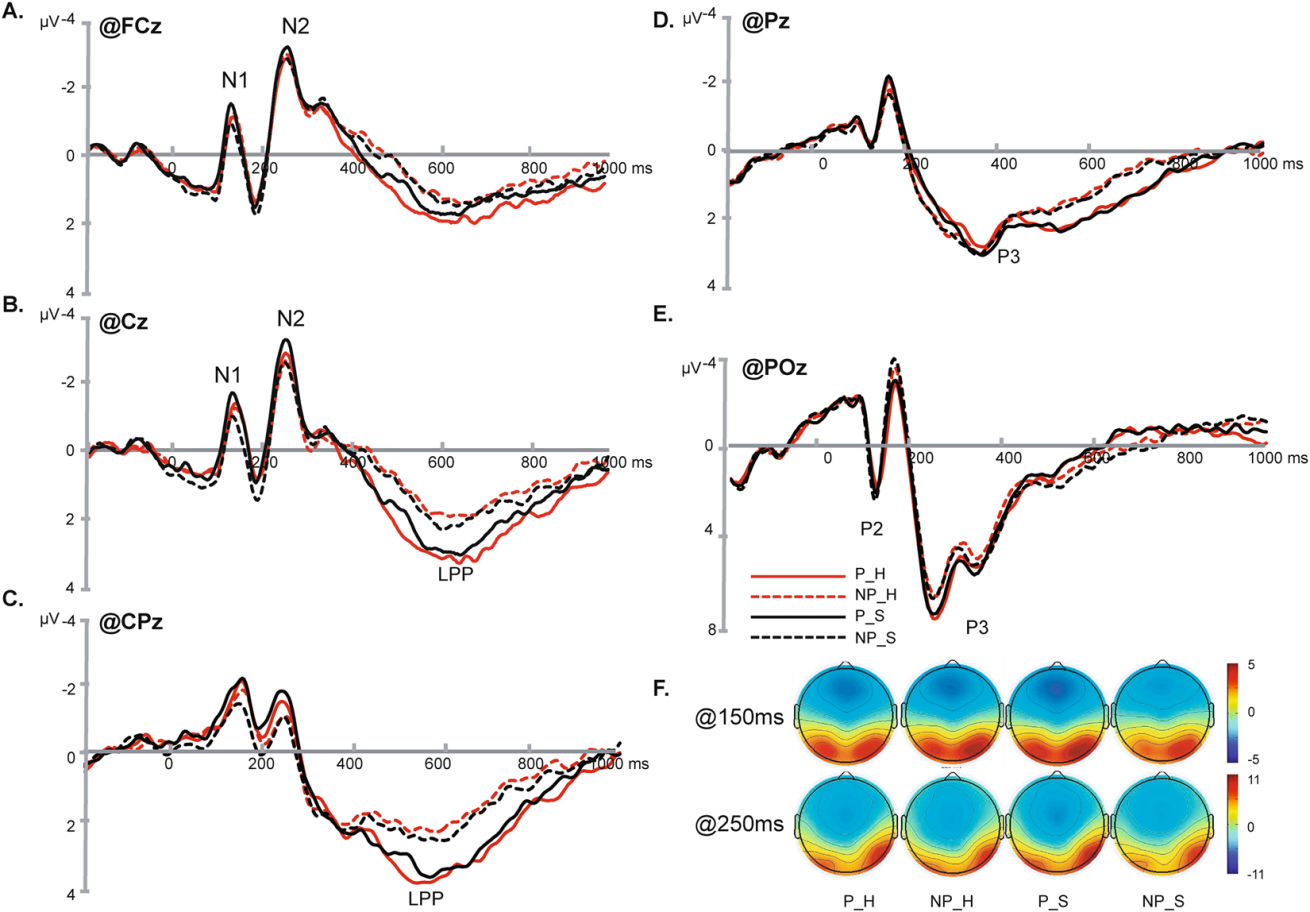
Grand average on FCz, Cz, CPz, Pz and POz (**A**–**E**) and the topographies at 150 ms and 250 ms (**F**) under all conditions.

**Figure 3:**
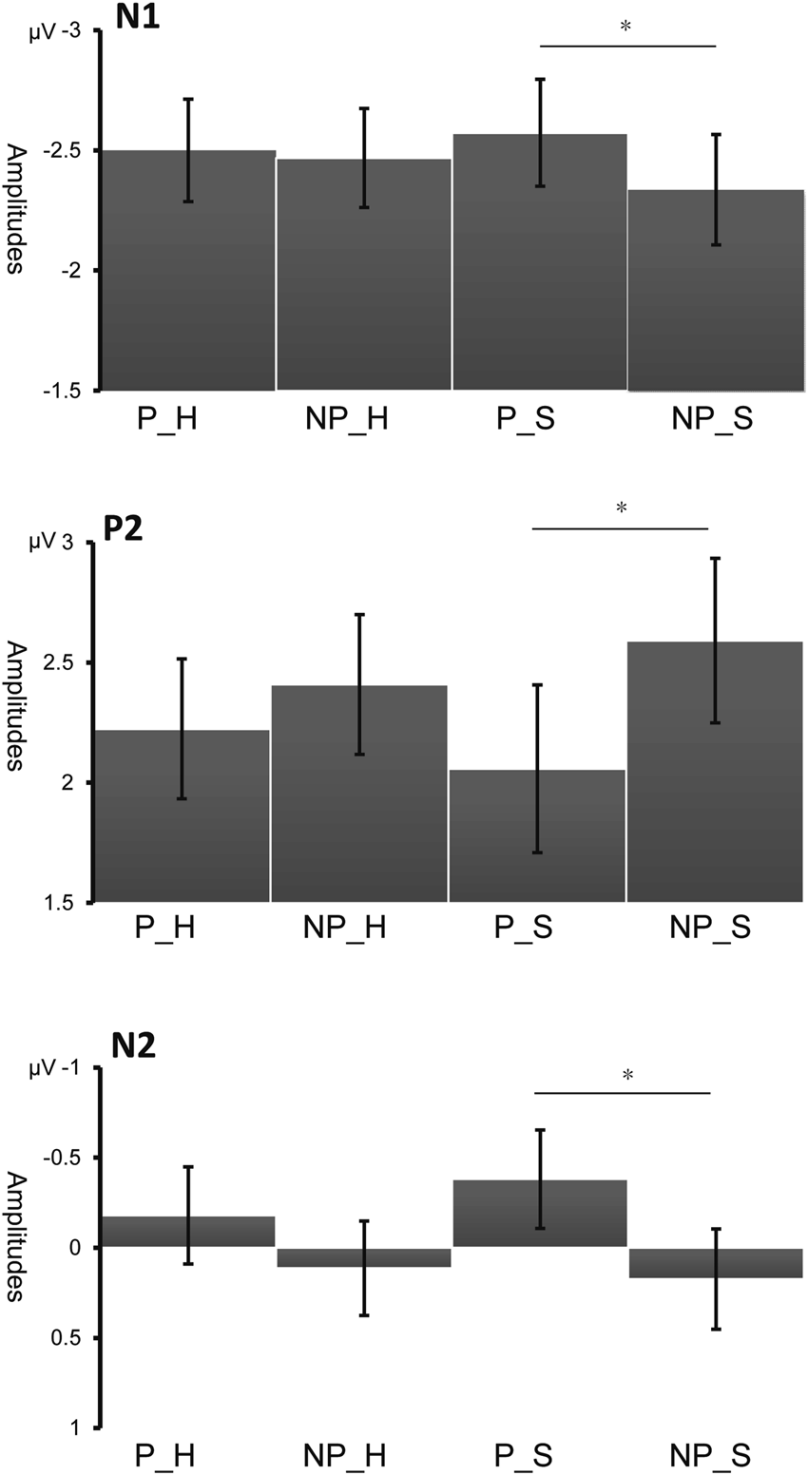
Interaction effects of N1, P2 and N2 components (mean ± S.E) (*p < 0.001).

The main effect of Mood was insignificant (F (1, 25) = 0.158, *p* = 0.695, η_p_^2^ = 0.006). The main effect of Picture was significant (F (1, 25) = 14.393, *p* = 0.001, η_p_^2^ = 0.365). Painful pictures elicited significantly larger amplitudes than non-painful pictures (−2.613 ± 0.208 µV and −2.042 ± 0.204 µV). The main effect of region was significant (F (4, 100) = 5.331, *p* = 0.021, η_p_^2^ = 0.176). N1 was mainly distributed in the frontal and central regions (−3.865 ± 0.721 µV, −3.196 ± 0.429 µV, −1.00 ± 0.178 µV, −1.725 ± 0.236 µV and −1.743 ± 0.351 µV). A significant interaction of Mood × Picture (F (1, 25) = 5.629, *p* = 0.026, η_p_^2^ = 0.184) was observed. Pairwise comparison showed that the painful picture elicited more negative amplitudes than the non-painful pictures only in the sad mood sessions (P_S: −2.5727 ± 0.222 µV, NP_S: −2.336 ± 0.230 µV, *p* < 0.001) but not in the happy mood sessions (P_H: −2.500 ± 0.213 µV, NP_H: −2.468 ± 0.206 µV, *p* = 0.755). Other interactions were insignificant (*ps* > 0.311) (Figs 2 and 3).

#### P2

The main effect of Mood was insignificant (F (1, 25) = 0.002, *p* = 0.963, η_p_^2^ < 0.001). The main effect of Picture was significant (F (1, 25) = 12.429, *p* < 0.001, η_p_^2^ = 0.450). Painful pictures elicited significantly smaller amplitudes than non-painful pictures (2.141 ± 0.309 µV and 2.500 ± 0.300 µV). The main effect of region was significant (F (4, 100) = 15.314, *p* < 0.001, η_p_^2^ = 0.380). P2 was mainly distributed in the parieto–occipital regions (1.773 ± 0.417 µV, 0.801 ± 0.321 µV, 0.282 ± 0.229 µV, 2.968 ± 0.705 µV and 5.770 ± 0.938 µV). A significant interaction of Mood × Picture (F (1, 25) = 4,536, *p* = 0.043, η_p_^2^ = 0.154). Pairwise comparison showed that the painful picture elicited smaller amplitudes than the non-painful pictures only in the sad mood session (P_S: 2.058 ± 0.349 µV, NP_S: 2.591 ± 0.342 µV, *p* < 0.001) but not in the happy mood session (P_H: 2.224 ± 0.291 µV, NP_H: 2.409 ± 0.291 µV, *p* = 0.140). Other interactions were insignificant (*ps* > 0.053) (Figs 2 and 3).

#### N2

The main effect of Mood was insignificant (F (1, 25) = 0.388, *p* = 0.953, η_p_^2^ = 0.584). The main effect of Picture was significant (F (1, 25) = 35.118, *p* < 0.001, η_p_^2^ = 0.450). Painful pictures elicited significantly more negative amplitudes than non-painful pictures (−0.280 ± 0.326 µV and 0.143 ± 0.264 µV). The main effect of region was significant (F (4, 100) = 31.686, *p* < 0.001, η_p_^2^ = 0.559). N2 was mainly distributed in the frontal and central regions (−3.301 ± 0.464 µV, −2.257 ± 0.354 µV, −0.472 ± 0.292 µV, 2.054 ± 0.622 µV and 3.633 ± 0.767 µV). A significant interaction of Mood × Picture (F (1, 25) = 7.169, *p* = 0.013, η_p_^2^ = 0.223) was observed. Pairwise comparison showed that the painful picture elicited larger N2 than the non-painful pictures only in the sad mood session (P_S: −0.381 ± 0.273 µV, NP_S: 0.174 ± 0.278 µV, *p* < 0.001) but not in the happy mood sessions (P_H: −0.180 ± 0.269 µV, NP_H: 0.113 ± 0.262 µV, *p* = 0.052). We also observed a significant interaction of Picture × Region (F (1, 25) = 4,174, *p* = 0.031, η_p_^2^ = 0.143). Pairwise comparison showed that the painful picture elicited smaller amplitudes than the non-painful pictures only in central, central-parietal, parietal and parieto–occipital regions (*p* = 0.043, *p* = 0.001, *p* < 0.001 and *p* = 0.001) but not in the frontal region (*p* = 0.807). The interaction of Mood × Region were insignificant (*p* = 0.797) (Figs 2 and 3).

#### P3

The main effect of Mood was insignificant (F (1, 25) = 0.229, *p* = 0.637, η_p_^2^ = 0.009). The main effect of Picture was significant (F (4, 100) = 4.831, *p* = 0.037, η_p_^2^ = 0.162). Painful pictures elicited significantly larger amplitudes than non-painful pictures (1.887 ± 0.216 µV and 1.718 ± 0.223 µV). The main effect of region was significant (F (4, 100) = 36.116, *p* < 0.001, η_p_^2^ = 0.591). P3 was mainly distributed in the parietal and parieto–occipital regions (−1.100 ± 0.287 µV, −0.063 ± 0.225 µV, 1.961 ± 0.264 µV, 3.910 ± 0.500 µV and 4.304 ± 0.640 µV). Other interactions were insignificant (*ps* > 0.206).

#### LPP

The main effect of Mood was insignificant (F (1, 25) = 0.066, *p* = 0.800, η_p_^2^ = 0.003). The main effect of Picture was significant (F (4, 100) = 99.187, *p* < 0.001, η_p_^2^ = 0.799). Painful pictures elicited significantly larger amplitudes than non-painful pictures (1.505 ± 0.189 µV and 0.888 ± 0.173 µV). The main effect of region was significant (F (4, 100) = 99.187, *p* < 0.001, η_p_^2^ = 0.799). LPP was mainly distributed in the central and central-parietal regions (0.977 ± 0.242 µV, 1.712 ± 0.144 µV, 1.906 ± 0.195 µV, 1.019 ± 0.390 µV and 0.166 ± 0.541 µV). A significant interaction of Picture × Region (F (1, 25) = 5.040, *p* = 0.029, η_p_^2^ = 0.168) was observed. Pairwise comparison showed that the painful picture elicited larger amplitudes than the non-painful pictures in the frontal, central, central-parietal and parietal regions (*p* = 0.001, *p* < 0.001, *p* < 0.001 and *p* < 0.001) but not in the parieto–occipital region (*p* = 0.058). Other interactions were insignificant (*ps* > 0.089).

## Discussion

In the present study, ERP responses to observing pictures showing other’s in painful or non-painful situations under a music-induced happy and sad mood were compared. We found significant interactions of Mood × Picture in components N1, P2, and N2 such as the painful pictures elicited significantly more negative amplitudes than the non-painful pictures only under the induced sad mood but not under the induced happy mood.

It should be noticed that in the literature of ERP studies in empathy for pain, many papers reported a positive shift of the painful condition comparing to the non-painful condition^11,13,39,44–46^. However, some studies reported an insignificant result in the early components (N1, P2 and N2); the positive shift was only observed in the later components such as P3 and LPP^13,47^. Besides, there are also studies reported a more negative shift in the early components and a more positive shift in the later components^14,48–50^. It is worthy to mention that different sets of stimuli were developed and used in different studies. Although we are not sure if this inconsistency was resulted by the differences between stimuli sets, it did imply that only using the amplitude of components to indicate the neural responses was not stable enough. We proposed to use the discrimination ability between painful and non-painful stimuli to indicate how well the stimuli were processed. If the painful and the non-painful stimuli were differentiated in one condition (difference of amplitudes (P-NP) was significant) but not in another condition, we can say the stimuli was better processed in the former condition. In the literature, this logic has been applied widely^11,48,49^.

The early component N1 has been suggested to be a marker of the automatic activation of affective arousal or emotional sharing^12,51^. The parieto-occipital P2 was typically elicited by visual stimuli and was suggested to represents some aspect of higher –order perceptual processing modulated by attention^52^. Larger P2 may indicate more attentional resources were allocated^53^. N2 was suggested to index an early automatic component related to the sensitivity to other’s pain, as well as a biomarker of the affective component of empathy for pain^54^. One ERP study found that N2 was enhanced when facing the pain of the participants’ own-race compared to other race. The author suggested the reduced N2 reflected suppressed affective responses towards other race’s pain in the early stage^14^. The amplitude of N2 was also found to be significantly correlated with a subjective rating of affective empathy and the participant’s scores in the Empathic Concern Scale^9^. In the current data, we found painful stimuli elicited significantly more negative N1, P2, and N2 than the non-painful stimuli only under the sad mood, but under the happy mood. These results indicated that people in a happy mood are less sensitive to other’s pain than people in a sad mood, and other’s pain gained more attention and was better processed in a sad mood. And the induced moods have an impact on the early, automatic stage of the process in empathy for pain.

Previous studies suggested that the amplitudes of P3 and LPP reflect motivational engagement and a commitment of attentional resources to the affective pictures^55,56^. Usually, stimuli that are more salient, arousing and motivational significant elicit larger amplitudes^57–61^. Consistent with these studies, in the current results, we found that the painful pictures elicited significantly larger P3 and LPP than the non-painful pictures in both happy and sad mood conditions. These results indicate that different moods did not influence the later, cognitive evaluation stage.

How can we explain this effect? One explanation we proposed was the “mood congruency hypothesis”. Mood manipulation has been correlated with shifts in attention to mood congruent stimuli: studies of inattentional blindness found that participants more often reported seeing unexpected mood congruent face-like stimuli vs. mood incongruent stimuli^62^. Previous studies suggest people are more tend to notice, to process and to memorize the stimuli that are congruent with their current moods. One extreme example comes from the enhanced negativity bias in depression^63–65^. Another study found positive affect can reduce people’s negative attentional bias and increase their positive attentional bias^66^. Other’s pain is one of the most salient and negative stimuli encountered in our real life, which can trigger unpleasant feelings, even somatosensory pain in the observers^4,67^. One ERP study using priming paradigm found that relative to non-painful pictures, differential P3 amplitudes for painful pictures were larger followed by negative primes than either neutral or positive primes. There were no significant differential P3 amplitudes for painful pictures relative to non-painful pictures followed neutral and positive emotional primes^38^. The author suggested that this finding indicated the negative emotional primes strengthen the observers’ attention toward others’ pain. These findings support that induced moods can bias people’s attention toward the stimuli that share the congruent emotion valence with their current mood. In the current design, the induced positive mood hampered the processing of negative stimuli around. Specifically, the participants were insensitive and less discriminative to other’s pain. However, due to a lack of neural control, we cannot measure whether the sad mood biased the processing of pain towards a better discrimination. Therefore, our results only partially support the “mood congruency hypothesis”.

There is also another possible explanation. Humans tend to use the self as a reference point to perceive the world and gain information about other people’s mental states. This self-referential projection mechanism can sometimes result in egocentrically biased judgments. The previous study proved that this bias exists in the affective domain (referred as emotional egocentricity bias (EEB)) and human beings are relied on the activities of right supramarginal gyrus to prevent this bias to go too far^68^. Numerous studies have shown that a positive mood can decrease pain perception in self^69^. For example, one study found induced sad and happy moods have differential effects on pain ratings in patients suffering from chronic back pain: comparing to induced sad mood, induced happy mood resulted in significantly lower pain ratings and higher pain tolerance^70^. Other studies reported that self-chosen, pleasant music can reduce the self-rated intensity of pain in Fibromyalgia^71,72^. When a person is in the happy mood, his/her own sensitivity to pain would be decreased. When they empathize with other’s pain, they are tending to use themselves as a basis to evaluate other’s pain^73^. Accordingly, the decreased sensitivity to pain in themselves might bias their judgment of other’s pain. Accordingly, in the happy mood, people tend to underestimate other’s pain.

According to Baston’s empathy-altruism hypothesis, the higher level of empathy should induce a higher level of prosocial behavior^74^, which seems to be contradictory to our findings that negative mood is associated with higher level of empathy for pain. Actually, there are previous studies that reported positive mood can be associated with prosocial behavior^75,76^. How to explain this contradictory result? A recent study found that it is the later component P3, but not the early components, that (i) signal intuitive prosocial motivation and (ii) predict subsequent engagement in prosocial behavior^77^. Previous studies also suggested that it was the cognitive component, but not the affective component of empathy that modulates the prosocial behaviors. An fMRI study reported that prosocial behavior is predicted by the brain regions (the dorsal part of the medial prefrontal cortex, dMPFC) related to the cognitive component of empathy^78^. In the current study, although the participants are insensitive to the other person’s pain in the early, automatic processing stage, they showed no difference in the later, top-down processing stage (P3 and LPP). The empathic response in the latter stage, reflecting in the later component P3 is the one that really matters to following prosocial behavior. The unaffected P3 component indicates that the cognitive component of empathy was not influenced by the induced moods. Therefore, although happy mood did weaken the early, automatic neural responses but not necessarily reduce prosocial behaviors. The measuring of the willingness of helping in the current study also supported that no difference was found between positive and negative moods.

In summary, the present study explored how the induced mood modulates the neural response to other’s pain. We found that compared to the induced negative mood (sad), the induced positive mood (happy) can reduce the early affective arousal, emotional sharing, and sensitivity to other’s pain, but not the later, cognitive evaluation process. The present finding indicates the observer’s current moods were crucial in the processing of other’s pain and when in a happy mood, people are less sensitive to other’s pain in the early processing stage.

There are two limitations existing in the current investigation. First of all, there was a lack of neutral control condition. This limitation causes the problem in explaining the results. In the literature, under neutral mood (no mood induction procedure in the experiment), a main effect of pain can be observed on N1 and N2 components very consistently. Under the happy mood, we failed to find the similar effects while under the sad mood, this effect persevered. Due to the lack of neutral control, we can only conclude that a happy mood does impair the sensitivity and affective sharing to other’s pain but we cannot conclude the exact effect of the sad mood. Secondly, we do not control the arousal level of happy and sad music inductions. The happy music excerpts were significantly more arousing than the sad ones. We know that the different emotions can be considered as the different combination of valence and arousal. For example, sadness is low arousal with negative affect; angry is high arousal with negative affect while happiness is high arousal with positive affect^78^. It is difficult to control the arousal level in the current design since we want to compare sad and happy moods. Therefore, it’s better to consider the “mood” induced by the music here as a combination of valence and arousal. And the effect observed was induced by the combination of valence and arousal instead of only one of them.
